# The essential peptidoglycan glycosyltransferase MurG forms a complex with proteins involved in lateral envelope growth as well as with proteins involved in cell division in *Escherichia coli*

**DOI:** 10.1111/j.1365-2958.2007.05851.x

**Published:** 2007-08

**Authors:** Tamimount Mohammadi, Aneta Karczmarek, Muriel Crouvoisier, Ahmed Bouhss, Dominique Mengin-Lecreulx, Tanneke den Blaauwen

**Affiliations:** 1 Molecular Cytology, Swammerdam Institute for Life Sciences, University of Amsterdam Kruislaan 316, 1098 SM Amsterdam, PO Box 194062, 1090 GB Amsterdam, the Netherlands; 2 Laboratoire des Enveloppes Bactériennes et Antibiotiques, CNRS, IBBMC UMR8619, Université Paris-Sud Bât. 430, 91405 Orsay, France

## Abstract

In *Escherichia coli* many enzymes including MurG are directly involved in the synthesis and assembly of peptidoglycan. MurG is an essential glycosyltransferase catalysing the last intracellular step of peptidoglycan synthesis. To elucidate its role during elongation and division events, localization of MurG using immunofluorescence microscopy was performed. MurG exhibited a random distribution in the cell envelope with a relatively higher intensity at the division site. This mid-cell localization was dependent on the presence of a mature divisome. Its localization in the lateral cell wall appeared to require the presence of MreCD. This could be indicative of a potential interaction between MurG and other proteins. Investigating this by immunoprecipitation revealed the association of MurG with MreB and MraY in the same protein complex. In view of this, the loss of rod shape of Δ*mreBCD* strain could be ascribed to the loss of MurG membrane localization. Consequently, this could prevent the localized supply of the lipid II precursor to the peptidoglycan synthesizing machinery involved in cell elongation. It is postulated that the involvement of MurG in the peptidoglycan synthesis concurs with two complexes, one implicated in cell elongation and the other in division. A model representing the first complex is proposed.

## Introduction

The characteristic shape of most bacteria is attributed to the peptidoglycan layer. This major constituent of the bacterial cell wall is responsible for the maintenance of the structural integrity of the cell by withstanding its internal turgor pressure. The biosynthesis of peptidoglycan is therefore essential and its role is unique to bacteria. As a consequence, enzymes involved in this complex process are used as targets for antibiotics. In *Escherichia coli* the assembly of peptidoglycan includes three phases (Ha *et al*., 2001). Phase one begins in the cytoplasm of the bacterial cell and involves the synthesis of UDP-*N*-acetylmuramyl-pentapeptide (UDP-MurNAc-pentapeptide) from UDP-*N*-acetyl-glucosamine (UDP-GlcNAc). During the second phase, which takes place on the cytoplasmic surface of the bacterial membrane, the activity of two essential enzymes, MraY and MurG, is required. MraY catalyses the transfer of the phospho-MurNAc-pentapeptide motif of UDP-MurNAc-pentapeptide to a lipid carrier, undecaprenyl phosphate, to form the so-called lipid I. MurG (a glycosyltransferase) then catalyses the transfer of the GlcNAc motif of UDP-GlcNAc to the C4 hydroxyl MurNAc in lipid I to produce the lipid-linked β-(1,4) disaccharide known as lipid II. In phase three, which is called cell surface stage, lipid II is translocated to the exterior surface of the cell by an unknown mechanism and incorporated into the peptidoglycan through transglycosylation and transpeptidation reactions by penicillin-binding proteins (PBPs) (Höltje, 1998; Nanninga, 1998; Vollmer and Höltje, 2001).

As MraY and MurG are key players in the biosynthesis of the peptidoglycan layer, studying these important enzymes could furnish insights into the development of inhibitors and subsequently to further understand the mechanism of the persisting problem of resistance to antibiotics. Up to now, MurG has been the subject of several studies. It has been shown that MurG is required for cell growth and survival (Mengin-Lecreulx *et al*., 1991) and that it is conserved among eubacteria. As it has no counterpart in mammalian cells, this enzyme is interesting to evaluate as a potential antibiotic target. Recently, the crystal structure was solved (Ha *et al*., 2000; Hu *et al*., 2003) and several (high-throughput) assays for screening inhibitors of MurG have been developed and validated (Auger *et al*., 2003; Helm *et al*., 2003; Liu *et al*., 2003; Zawadzke *et al*., 2003; Ravishankar *et al*., 2005; Ramachandran *et al*., 2006). Although these studies have elucidated the kinetic characterization of MurG, little is known about its mechanism of action at the level of morphogenesis and in particular its molecular interactions with other proteins.

Therefore, the aim of the present study was to provide a cellular characterization of MurG and to determine putative interactions with proteins of *E. coli*, in an attempt to elucidate its specific function in relation to cell elongation and cell division. To this end, immunofluorescence microscopy (IFM) and immunoprecipitation (IP) were applied. MurG appeared to be randomly distributed in the cell envelope with a relatively higher intensity at the division site. This mid-cell localization was dependent on the presence of the divisome. Particularly, for this position MurG required the presence of PBP3 and FtsQ. Furthermore, the results of IP using chemical cross-linking revealed the association of MurG with MreB and MraY in the same protein complex. The localization of MurG appeared also to be dependent on the expression of the *mreBCD* operon. This suggests that the defect in cell elongation caused by the absence of MreBCD is possibly due to the inability of MurG to position the lipid II peptidoglycan precursor.

## Results

### MurG is tightly associated with the cytoplasmic membrane

Outer and inner membranes of inside-out membrane vesicles were separated using sucrose density equilibrium centrifugation and the presence of MurG was assessed by immunoblotting using antibodies raised against this protein. MurG was shown to be present in the inner membrane fraction [inner membrane vesicles (IMVs)] corresponding to the 45% sucrose layer. This fraction was further used to study the characteristics of interaction of MurG with the inner membrane. As shown in Fig. S1, association of MurG with the inner membrane was not affected by chemical reagents such as 2 M NaCl, and 0.1 M Na_2_CO_3_, whereas 50 mM EDTA and 6 M urea caused a slight decrease in the intensity of the protein band. This implies that MurG is strongly associated with the inner membrane. However, MurG could be released from the inner membrane after treatment with 1% Triton X-100 as reported (Van den Brink-van der Laan *et al*., 2003) and upon subjection to trypsin digestion (Fig. S1). These results suggest that the interaction of MurG with the cytoplasmic membrane is predominantly of hydrophobic nature.

### MurG localizes in the lateral cell wall as well as at the division site

The number of MurG molecules per average cell present in cell extracts of *E. coli* LMC500 (wild-type K-12) grown to steady state at 28°C in GB1 medium was determined by immunoblotting and densitometrical comparison of the MurG signal from the cell extract (total lysate) with that of a concentration gradient of purified MurG. MurG was estimated to be present in 1200 ± 33 (*n* = 3) molecules per average cell (see Fig. S2).

**Fig. 1:**
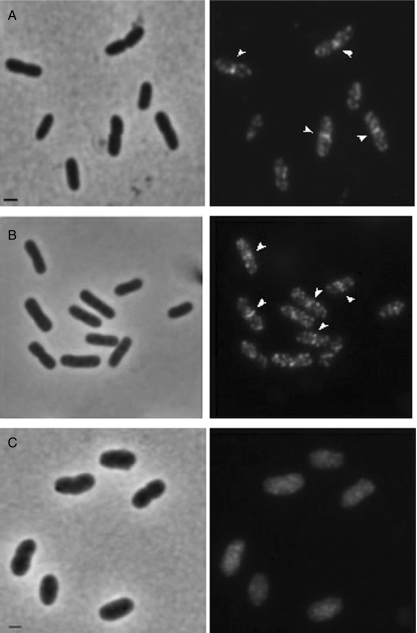
Localization of MurG in wild-type *E. coli* LMC500 (A) and in the MurG(Ts) strain GS58 (B and C). Phase-contrast images are given on the left and fluorescence images on the right. LMC500 cells were grown to steady state at 28°C in GB1 (A), fixed, permeabilized and immunolabelled with anti-MurG. GS58 cells were first grown to mid-exponential phase at 28°C in TY medium (B) shifted to 42°C and allowed to grow for 2 MDs (C). Thereafter they were fixed, permeabilized and subjected to MurG IFM. The arrows point to the band at mid-cell. All panels have the same exposure time. Scale bar equals 1 μm.

**Fig. 2:**
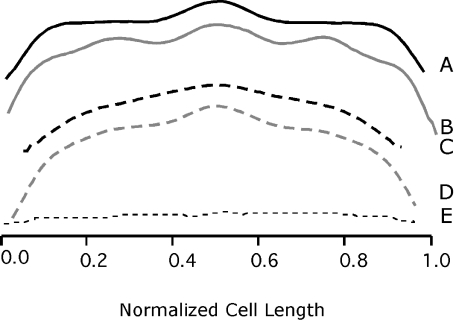
Fluorescence intensity profiles of LMC500 cells grown at 28°C in TY (A and B) or in GB1 (C and D) and immunolabelled with anti-MurG. The average normalized cell length is given on the *x*-axis. The average normalized fluorescence intensity (AU) is reflected on the *y*-axis. Fluorescence intensity was determined in cells (*n* ≥ 500) that have no visible constriction (A and C) and in constricting cells (B and D). To indicate the background fluorescence (unspecific binding of secondary antibody), the immunolabelling procedure was carried out with LMC500 cells where the incubation with the first antibody (anti-MurG) was omitted (E). Profiles were measured using Object-image as described in *Experimental procedures*.

Cellular localization of MurG in LMC500 grown at 28°C in GB1 and TY was examined by IFM. MurG localized as multiple distinct foci in the cell envelope (Fig. 1A). Because MurG was mostly present in the inner membrane but almost absent in the cytosol as shown by immunoblotting (see Fig. S3), the foci must represent envelope-associated MurG. Analysis of the normalized average fluorescence intensity profile (FIP) of a total of more than 500 LMC500 cells revealed a random fluorescence distribution of MurG in the cell envelope with a relatively higher intensity at the division site (Fig. 2). The intensity increase at mid-cell was not a result of the constriction, i.e. doubling of membranes (compare with the FIP of a general lipid stain of the membranes with BODIPY-C12 in fig. 8A of Bertsche *et al*., 2006). In addition to this mid-cell localization of MurG in TY-grown cells, a denser distribution at predivision sites (i.e. 1/4 and 3/4 positions) of the future daughter cells (Fig. 2B) was visible. This is consistent with the proposition of random incorporation of precursors of peptidoglycan in *E. coli* during lateral growth with a higher synthetic activity at the middle of the cell before a constriction was visible (Taschner *et al*., 1988b; Wientjes and Nanninga, 1989).

**Fig. 8:**
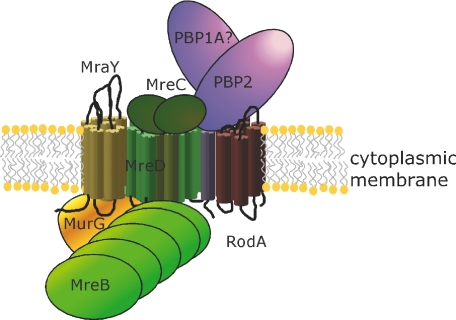
Schematic representation of the possible constituents of the putative cell elongation complex in *E. coli*. MraY, MurG and MreB interact with the MreCD. The complex of MreBCD, PBP2 and RodA was published by Kruse *et al*. (2005). As MreC was shown to interact with three of the four high-molecular-weight class A PBPs of *B. subtilis* (Van den Ent *et al*., 2006), it was postulated that PBP1A could be part of the complex (see *Discussion*). For simplicity only one MurG molecule is illustrated. However, the possibility of formation of dimmers that might be part of the complex is not excluded.

The cellular localization of MurG was also studied in the MurG temperature-sensitive (Ts) strain GS58 (Salmond *et al*., 1980), which expresses a non-functional truncated MurG (amino acids 1–290) at 42°C due to an amber mutation at W290 (at the restrictive temperature a STOP replaces the W290 tryptophan present in the wild-type strain (Crouvoisier *et al*. to be published elsewhere)). These cells grow well at 28°C in TY and show a normal MurG localization pattern (Fig. 1B) but after a shift to 42°C for 2 mass doublings (MDs) they become abnormally wide and swollen and finally they start to lyse. At this restrictive temperature, MurG was evenly distributed in the cytoplasm and mid-cell localization was not detectable (Fig. 1C). The non-functional truncated MurG could be detected by the used antibody when immunoblotting was performed. The absence of membrane localization was therefore not due to insufficient recognition of MurG by the antibody. This indicates that the carboxy-terminal 66 amino acids of MurG are essential for its membrane localization either because they may interact with membrane components or because their absence may affect the folding of MurG.

### Mid-cell localization of MurG requires divisome components

**Fig. 3:**
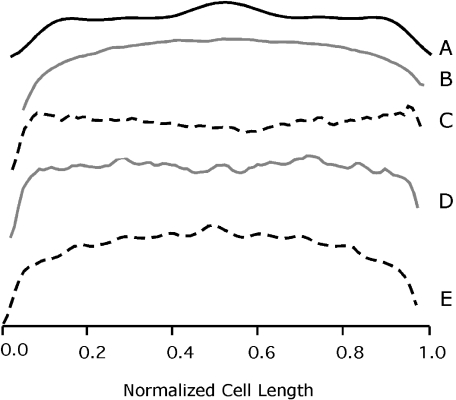
Fluorescence intensity profiles of LMC509 (FtsZ(Ts), A and B), LMC510 (PBP3(Ts), C), LMC531 (FtsQ(Ts), D) and LMC500 (wild-type, E) cells. After growth at 28°C in 1/2 GB1 LMC509 cells were shifted to 42°C for 2 MDs, and immunolabelled with anti-MurG (A, 28°C) and (B, 42°C). LMC510 and LMC531 cells were grown at 28°C in GB1 to steady state and shifted to 42°C for 2 MDs, and immunolabelled with anti-MurG. LMC500 cells were grown at 28°C in GB1 after which aztreonam was added and growth was continued for 2 MDs before the cells were immunolabelled with anti-MurG (E). Similar patterns shown in A were also obtained for the other temperature-sensitive strains when grown at 28°C in GB1. The average normalized cell length is given on the *x*-axis. The *y*-axis represents the average normalized fluorescence intensity.

To investigate whether the presence of MurG at mid-cell was division dependent, its localization was studied in the strain LMC509 that expresses the temperature-sensitive FtsZ84(Ts) protein, which is not able to polymerize at mid-cell and causes filamentation at the restrictive temperature (Addinall *et al*., 1997). MurG showed normal mid-cell localization in this strain at 28°C (Fig. 3A). However, at 42°C MurG foci were randomly distributed in the cell envelope (Fig. 3B). This suggests that the mid-cell localization of MurG is related to cell division. The earliest event in cell division is the assembly of the FtsZ-ring. This is followed by the assembly of a number of other cell division proteins at mid-cell about 1/5 of a generation time later just before the onset of the constriction process (Aarsman *et al*., 2005). To examine whether the MurG mid-cell localization is dependent on an early or a late event in the process of cell division, localization studies of MurG were further performed in strain LMC510, which contains the *ftsI2158* that expresses a PBP3(Ts) protein. PBP3 is involved in peptidoglycan synthesis and localizes to the division site during the later stages of cell growth and throughout septation (Weiss *et al*., 1999; Aarsman *et al*., 2005). At 42°C the PBP3(Ts) filaments showed blunt constriction where mid-cell localization of MurG was not observed (Fig. 3C and Fig. S4A). This implies that the position of MurG is dependent on the presence of PBP3.

For its recruitment to the division site it was described in several studies (reviewed in Errington *et al*., 2003) that PBP3 requires the presence of other proteins (i.e. FtsL, FtsB, FtsQ and FtsW). FtsQ localization is believed to be indispensable for the recruitment to the divisome of the majority of the late localizing proteins. To verify whether it is also important for the positioning of MurG, localization of this latter was examined in the temperature-sensitive LMC531 strain that expresses FtsQ(Ts) in which glutamic acid (E) 125 is replaced by a lysine (K) (Storts and Markovits, 1991). At the restrictive temperature, immunolabelling of the LMC531 filaments with anti-MurG revealed the absence of MurG at 1/4 and 3/4 positions. Besides, a dense fluorescent signal at mid-cell was not visible (Fig. S4B) [absence of MurG mid-cell localization was also observed in the FtsW(Ts) strain (data not shown)]. This indicates that the position of MurG depends on the localization of FtsQ and evidently on a mature divisome. To gain more insight in the nature of this reliance, MurG localization in LMC500 was determined in the presence of aztreonam, a specific inhibitor of PBP3. This was undertaken to find out whether the appearance of MurG required the function of PBP3. Mid-cell localization of MurG was preserved in aztreonam-induced filaments after 2 MDs (Fig. 3E see the distinct peak at mid-cell, and Fig. S4C). As reported previously (Den Blaauwen *et al*., 2003), most cell division proteins (including PBP3) are able to localize at mid-cell in the presence of aztreonam for at least 2 MDs, whereas the localization at future division sites at 1/4 or 3/4 positions is inhibited. MurG followed this pattern suggesting that the presence of MurG at mid-cell takes place even if the divisome is not functional.

### MurG associates with MreB in a protein complex

As alluded to above MurG seems to form a complex with proteins that are part of the divisome. Therefore, it is possible that it could also interact, because of its localization in the cell envelope with proteins involved in lateral envelope synthesis. To assess this, IP experiments were carried out.

**Fig. 4:**
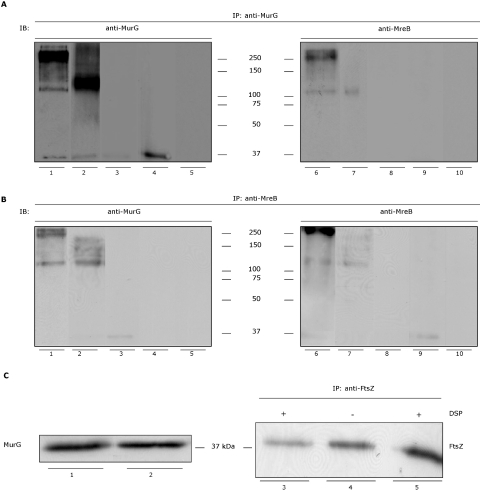
Immunoblotting (IB) analysis of the protein complex with anti-MurG and anti-MreB. IP was performed on Triton X-100 solubilized membranes prepared from wild-type *E. coli* cells (LMC500), the MraY–β-lactamase–His fusion protein expression strain BW25113Δ*mraY*/pMAK*mraY*ec, the Δ*mreBCD* mutant PA340-678 cells, the MurG(Ts) strain GS58, and cross-linked with DSP. The detection was carried out with anti-MurG (A, lanes 1–5, and B, lanes 1–5) or anti-MreB (A, lanes 6–10, and B, lanes 6–10). The protein complex is visible among a smeared background in LMC500 (A, lanes 1 and 6). The dominant bands of this complex have a molecular weight of about 120 kDa and approximately 250 kDa. In the strain BW25113Δ*mraY*/pMAK*mraY*ec (A, lane 2) the 120 kDa band is more pronounced. The band at the position of 37 kDa is free MurG. No protein complex was yielded in the control where the IP procedure was carried out without the membrane fractions (A, lanes 5 and 10). The same complexes (of 250 kDa and 120 kDa) are also seen when the immunoblot was probed with anti-MreB (A, lanes 6 and 7 respectively). In the strain Δ*mreBCD* PA340-678 the protein complex is absent (A, lanes 3 and 8) and only free MurG is visible at the level of about 37 kDa when the detection is carried out with anti-MurG (A, lane 3). The complexes of 250 kDa and 120 kDa were undetectable in the MurG(Ts) strain GS58 (A, lanes 4 and 9). The protein band of about 37 kDa is free MurG. Performing the IP with anti-MreB resulted as well in a protein complex of approximately 250 kDa and 120 kDa in LMC500 when probing with anti-MurG or anti-MreB (B, lanes 1 and 6 respectively). The 120 kDa band was more pronounced in the strain BW25113Δ*mraY*/pMAK*mraYec* (B, lanes 2 and 7). The complex was not visible in the Δ*mreBCD* PA340-678 cells (B, lanes 3 and 8). In this strain only a MurG band at the position of 37 kDa was detected when probing with anti-MurG (B, lane 3). In the MurG(Ts) the protein complex was absent (B, lanes 4 and 9). A faint MreB band is visible when anti-MreB was used for detection (B, lane 9). To assess background binding of the secondary antibodies the incubation with the primary antibodies was omitted from the procedure. As depicted in Fig. S5 (see *Supplementary materials*) no protein bands are detected. One tenth of the membrane fraction used for the IP experiments was applied (without cross-linking) on SDS-PAGE and probed with anti-MurG (C, lanes 1 and 2). In LMC500 and BW25113Δ*mraY*/pMAK*mraYec* the intensity of the band (corresponding to MurG) is comparable (lanes 1 and 2 respectively). The interaction between MurG and MreB is specific. To show this the IP procedure with the membrane fraction was performed with anti-FtsZ and the immunoblot was probed with anti-FtsZ. Only a band (∼40 kDa) at the position of FtsZ is detectable in the presence (lane 3) and absence (lane 4) of DSP under non-reducing (lanes 3 and 4) or reducing (lane 5) conditions. The molecular weight of MurG, MreB and MraY–β-lactamase–His is 37.8, 36.9 and 71 kDa respectively. The data are representative of at least three separate experiments. Although some samples were not run in the same gel, the IB procedure (i.e. electrophoresis, blotting and ECL detection conditions) was the same for all of them.

Membrane fractions prepared from *E. coli* LMC500 strain grown at 28°C in GB1 or at 37°C in TY were Triton X-100 solubilized, chemically cross-linked with the membrane-permeable dithiobis (succinimidyl propionate) (DSP), immunoprecipitated using affinity-purified anti-MurG IgG-coated magnetic beads, and analysed by immunoblotting using anti-MurG. Under non-reducing conditions (sample buffer without β-mercaptoethanol, incubation at 37°C for 5 min before electrophoresis), the results showed that MurG was cross-linked into a high-molecular-weight complex. This protein complex is visible among a smeared background in wild-type LMC500 (Fig. 4A, lane 1). The dominant bands of this complex have a molecular weight of approximately 250 kDa and 120 kDa (Fig. 4A, lane 1). The band at the position of about 37 kDa is free MurG (that is not completely cross-linked in the complex). To ensure use of equal amounts of materials, a fraction of the membranes was loaded on SDS-PAGE and analysed with anti-MurG. The blot depicted in Fig. 4 (lanes 1 and 2) represents 1/10 of the membrane fraction used for the IP.

Complexes at the position of 250 kDa and around 120 kDa were likewise obtained when the immunoblot was probed with anti-MreB (Fig. 4A, lanes 1 and 2). Conducting the IP procedure with magnetic beads coated with affinity-purified anti-MreB yielded the presence of a protein complex as detected with anti-MurG and anti-MreB with a comparable high molecular weight as well (Fig. 4B, lanes 1, 2, 6 and 7), indicating that the MurG-containing protein complex could also be immunoprecipitated with MreB.

To exclude the possibility that the presence of the 250 kDa protein complex could be the result of cross-reactivity of the secondary antibodies with IgG from the beads, the IP procedure was conducted without the addition of the membrane fraction. As depicted in Fig. 4 (A: lanes 5 and 10, B: lanes 5 and 10) immunoblotting with anti-MurG or anti-MreB, respectively, did not yield any product. In addition, controls consisting of incubating the blots with only the secondary antibody to monitor the potential presence of IgG on the blot confirmed the absence of protein bands and hence background binding (Fig. S5A and B). Moreover, the complex was absent when IP was performed with either anti-MurG or anti-MreB of the membrane fraction of the *mreBCD* deletion strain PA340-678 (Fig. 4A, lanes 3 and 8, Fig. 4B, lanes 3 and 8) in which MurG is also cytosolic (see next section and Fig. S3) and of the MurG(Ts) strain GS58 (Fig. 4A, lanes 4 and 9, Fig. 4B, lanes 4 and 9). Taken together, the results provide evidence for association of MurG and MreB in the same protein complex at the periphery of the membrane of *E. coli*.

To assess whether this interaction was specific, the IP procedure with the membrane fraction was carried out with anti-FtsZ and the immunoblot was probed with anti-FtsZ. Although FtsZ is thought to be predominantly a cytosolic protein, Koppelman *et al.* (2004) have demonstrated that inside-out cytoplasmic membrane vesicles (IMV) contain a fraction of cytoplasmic membrane-bound FtsZ (IMV–FtsZ). The results of this IP revealed the presence of only one band of ∼40 kDa at the position of FtsZ (Fig. 4C, lanes 3–5). This band was observed in the presence (Fig. 4C, lane 3), and absence (Fig. 4C, lane 4) of the cross-linker under non-reducing (lanes 3 and 4) or reducing (incubation at 100°C for 10 min in sample buffer containing β-mercaptoethanol before electrophoresis) conditions (lane 5). Hence, the absence of high-molecular-weight complexes could be explained by the effect of treatment with Triton X-100 that may have disrupted all interactions of FtsZ with cell division proteins and also a potential association of MurG with FtsZ (as MurG was not detected). Additional control experiments were also performed involving IP with anti-PBP1B. PBP1B is a bitopic membrane protein that catalyses the transglycosylation and transpeptidation of precursors in peptidoglycan synthesis (Höltje, 1998; Vollmer and Höltje, 2001). Western blot analysis of the IP did not yield any protein complex containing MurG or MreB (results not shown). Based on the above described IP experiments and controls it can be concluded that the association of MurG with MreB was specific.

### MurG associates with MraY in a protein complex

MraY catalyses the transfer of the phospho-MurNAc-pentapeptide motif from UDP-MurNAc-pentapeptide to undecaprenyl phosphate leading to the formation of undecaprenyl-pyrophospho-MurNAc-(pentapeptide) (lipid I). MurG then transfers the GlcNAc motif from UDP-GlcNAc onto the lipid I, forming the disaccharide derivative undecaprenyl-pyrophospho-GlcNAc-MurNAc-(pentapeptide) (lipid II) (Bouhss *et al*., 2004). Lipid II is then polymerized and cross-linked into peptidoglycan by transglycosylation and transpeptidation reactions of the PBPs. As MraY and MurG are consecutively involved in the same biosynthetic pathway, it was reasoned that an interaction between these two proteins could exist. To investigate this assumption IP was performed with membrane fractions prepared from the strain BW25113Δ*mraY*/pMAK*mraY*ec using affinity-purified anti-MurG. In this strain the endogenous *mraY* gene is deleted and complemented by an MraY–β-lactamase–His fusion protein expressed from plasmid. This strain was used due to the lack of antibodies directed against MraY.

**Fig. 5:**
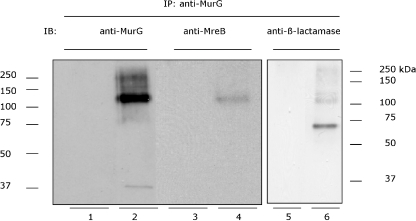
Immunoblotting (IB) analysis of the protein complex with anti-MurG, anti-MreB and anti-β-lactamase under non-reducing conditions. The membranes extracted from BW25113Δ*mraY*/pMAK*mraY*ec strain (MraY–β-lactamase–His expressing strain) were used for the IP (with cross-linking) with anti-MurG. The blot was then probed with anti-MurG (lanes 1 and 2), anti-MreB (lanes 3 and 4) or anti β-lactamase (lanes 5 and 6). In parallel the same IP was performed without the membrane fraction (lanes 1, 3 and 5). A cross-linked product with a molecular weight of about 120 kDa is visible in all samples except in control samples (lanes 1, 3 and 5). The faint band in lane 2 is MurG, which is not completely cross-linked in the protein complex. The band in lane 6 with the molecular weight of approximately 70 kDa corresponds to the MraY–β-lactamase–His protein that is not completely cross-linked in the protein complex.

Probing the immunoblot with anti-MurG resulted in cross-linked products among a smeared background. The prominent protein band of these products had a size of about 120 kDa (Fig. 4A, lane 2 and Fig. 5, lane 2). This band was also detected with anti-MreB (Fig. 4, lane 2 and Fig. 5, lane 4). Performing the analysis with a polyclonal antibody directed against β-lactamase (to detect the MraY of *E. coli* in fusion with β-lactamase) revealed the presence of a band with a molecular weight of around 120 kDa in addition to a 70 kDa band that corresponded to the molecular weight of MraY–β-lactamase–His fusion (Fig. 5, lane 6). As the complex was analysed under non-reducing conditions on SDS-PAGE, it might be of importance to mention that the molecular weight of the complexes might slightly diverge from the genuine mass of the separate proteins forming the complex. The 250 kDa complex could also be detected although the intensity of this band is much less than the cross-linked product in wild-type LMC500 strain and almost indistinguishable from the background. This difference might be attributed to the fact that LMC500 and BW25113Δ*mraY*/pMAK*mraY*ec are distinct strains. Control experiments where the same IP procedure was carried out without the membrane fractions yielded no products (Fig. 5, lanes 1, 3 and 5) indicating that MurG and MraY interact. As a whole these findings suggest that MurG, MreB and MraY were cross-linked in the same protein complex.

### Localization of MreB does not require the presence of MurG

Interaction of MurG and MreB also raised the question whether they are dependent on each other for their subcellular localization. To elucidate this, the following IFM experiments were conducted.

Prior to performing localization studies of MreB with IFM, the presence of MreB on the IMVs was assayed following the same procedure as aforementioned for MurG. Using affinity-purified anti-MreB antibody, the presence of MreB was detected mainly in the cytosol and in the inner membrane fractions. Unlike MurG, MreB could be extracted from the IMVs after treatment with 6 M urea or 0.1% Triton X-100 but not with 50 mM EDTA, 2 M NaCl or 0.1 M Na_2_CO_3_ (data not shown), pointing to an association of this protein with the inner membrane.

**Fig. 6:**
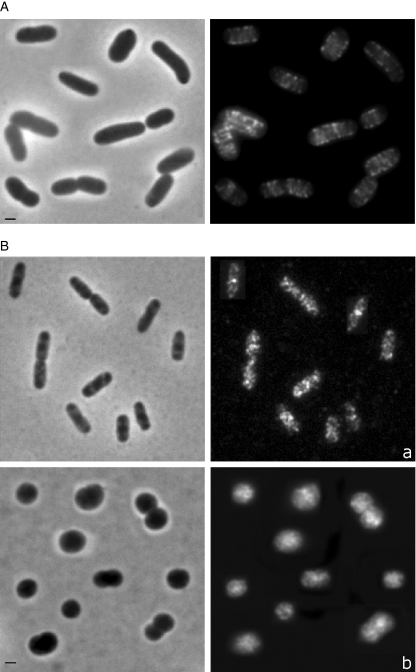
Localization of MreB in the MurG(Ts) strain GS58 grown at the restrictive temperature (42°C) in TY for 2 MDs (A). The MreB helical structure was maintained in the absence of the functional full-length MurG protein. (See for MurG localization in these cells Fig. 1C.) MurG localization in the absence of MreB helix (B). IFM was performed with PA340 (wild type) and PA340-678 (Δ*mreBCD*) cells grown at 28°C in TY to mid-exponential phase. MurG localizes as multiple foci in the cell envelope and at mid-cell in the wild-type strain (a) and is evenly distributed in the cytoplasm with no clear mid-cell localization in the Δ*mreBCD* strain, although a packed localization is visible (b). Phase contrast (left) and fluorescence images (right) are shown. The image contains a collection of representative cell. Scale bar equals 1 μm.

In wild-type K-12 strains MreB forms a regular, dynamic helix lying underneath the cytoplasmic membrane (Shih *et al*., 2005). Visualization of MreB with IFM was carried out in the MurG(Ts) strain GS58 to find out whether its localization relies on the presence of functional MurG. Cells grown at 28°C in TY displayed normal rod-shape and MreB localized in a helical pattern as was observed before (results not shown). After growth at 42°C for 2 MDs, the helical structure was preserved regardless of the aberrant shape of the cells (Fig. 6A). Apparently, localization of MreB does not require the presence of a functional MurG.

### Is localization of MurG dependent on the presence of MreB?

Conversely, to demonstrate whether localization of MurG is dependent on the presence of MreB, IFM experiments targeting MurG were performed with the strain PA340-678. This strain possesses a spherical morphology due to the deletion of the *mreBCD* operon (Wachi *et al*., 1987). MreB localizes in a helical pattern in the parental strain PA340, but is absent in the Δ*mreBCD* deletion strain PA340-678 (see Fig. S6). Interestingly, the localization of MurG was cytosolic (Fig. 6B, b) like the localization pattern observed in the MurG temperature-sensitive strain GS58 (Fig. 1C). In addition, a discernible packed (arcked filamentous) localization of MurG in constricting cells at the position of the future constriction sites was also observed. Apparently, this could reflect the presence of MurG that is part of the divisome and required by the division process in these cells.

**Fig. 7:**
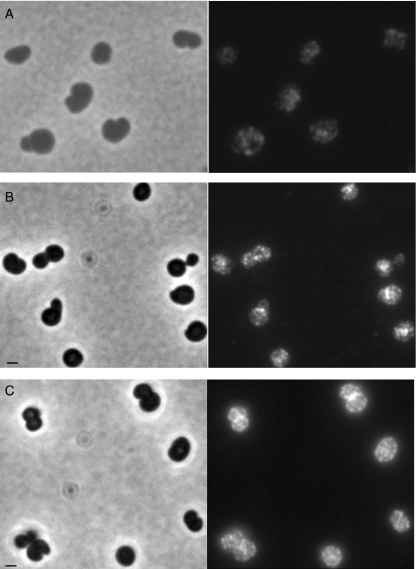
MurG and MreB localization are independent of the spherical cell morphology (A and B). LMC500 cells were grown for 2 MDs in the presence of mecillinam (inhibitor of PBP2), at 28°C in GB1. Cells were immunolabelled with anti-MreB (A) or anti-MurG (B). The MreB helical structure is preserved and the multi-foci localization pattern of MurG is observed. Arrangement of MurG is dependent on the presence of MreCD (C). IFM was performed with PA340-678pMEW1 strain (*mreBCD* deletion strain with the pMEW1 plasmid that expresses MreC and MreD constitutively) grown at 28°C in TY to mid-exponential phase. In these spherical cells MurG localized normally as multiple foci in the cell envelope and at mid-cell. Phase contrast (left) and fluorescence images (right) are shown. Scale bar equals 1 μm.

In the parental wild-type strain PA340 MurG exhibited a normal localization as multiple foci in the cell envelope (Fig. 6B, a). As PA340-678 cells are round, it is possible that the observed abnormality in MurG localization pattern was due to the aberrant cell morphology rather than to the absence of the MreB helix. To test whether the localization of MurG would be maintained in spherical cells exhibiting a helical arrangement pattern of MreB, LMC500 cells were grown for 2 MDs in the presence of 2 μg ml^−1^ of the PBP2 inhibitor mecillinam (Park and Burman *et al*., 1973). A functional PBP2 is essential for length growth and maintenance of the diameter of the cells (Den Blaauwen *et al*., 2003). Therefore inhibition of PBP2 by its specific inhibitor mecillinam results in spherical growth. The round cells were subsequently labelled with anti-MurG and anti-MreB. Both patterns of MreB and MurG localization were similar to those observed in rod-shape wild-type *E. coli* (Fig. 7A and B respectively, see also Fig. S7). This indicates that MurG does not require a rod-shape cell morphology for a proper localization, and that cytosolic localization visualized in round Δ*mreBCD* cells is most likely due to the absence of the Mre proteins rather than to the altered cell morphology.

To determine whether the absence of the MreB helix was responsible for the anomalous MurG localization, IFM experiments with the strain PA340-678 pMEW1 (harbouring the pMEW1 plasmid that expresses MreC and MreD constitutively) was performed. This revealed anormal localization (random distribution and at mid-cell) of MurG (Fig. 7C). To provide more evidence for this observation, localization of MurG was examined in wild-type *E. coli* treated with A22. This molecule was described to be a specific inhibitor of MreB (Iwai *et al*., 2002). It was also reported that addition of A22 induces spherical cell form by inhibiting cell elongation (Iwai *et al*., 2004). MreB is not able to localize in these cells (Karczmarek *et al*., 2007). The arrangement of MurG in *E. coli* cells treated with 40 μg ml^−1^ of A22 for 2 and 3 MDs was not altered (data not shown), denoting that its arrangement is dependent on the presence of MreC and MreD rather than on the presence of MreB. This is consistent with the results published very recently, while revising this manuscript (Aaron *et al*., 2007) where the cell wall synthesis in *Caulobacter crescentus* was studied with special emphasis to the role of FtsZ, MurG and MreB. The authors demonstrated that MurG accumulated near mid-cell. This accumulation took place shortly after the formation of the FtsZ ring and before constriction. This underlines a high synthesis and thereby availability of lipid II peptidoglycan precursors at the FtsZ ring location. It was also shown that mid-cell accumulation of MurG depended on FtsZ but not on MreB or the presence of substrate.

## Discussion

The present work was conducted in an attempt to get more insights in the specific function of MurG in relation to cell elongation and cell division. Particularly, a cellular characterization of MurG was conducted and its putative interactions with other proteins of *E. coli* were studied.

### Subcellular localization of MurG

MurG catalyses the last intracellular step of peptidoglycan synthesis (Mengin-Lecreulx *et al*., 1991). This glycosyltransferase was reported to be associated with the cytoplasmic face of the inner membrane (Bupp and Heijenoort, 1993; van den Brink-van der Laan *et al*., 2003). As presented here, this interaction was not or slightly affected by the presence of high salts, alkaline conditions, chelating agents or strong reagents. The association of MurG with the inner membrane could only be disrupted after treatment with 1% Triton X-100 or trypsin, indicating that it is predominantly of hydrophobic nature. This is in accordance with the proposal of Hu *et al*. (2003), who deduced from the crystal structure of MurG that the membrane association site of this protein consists of a hydrophobic patch in the N-domain surrounded by basic residues.

Immunofluorescence microscopy studies demonstrated that MurG was randomly distributed in the cell envelope with a higher intensity at the division site. The concentrated presence of MurG at the division site supports the results published earlier by Wientjes and Nanninga (1989). Using two different and independent approaches, i.e. pulse-labelling of synchronized cultures and autoradiography of pulsed cells, the authors showed an increased localized peptidoglycan synthesis activity at the (potential) division sites. Based on immunodetection and electron microscopy techniques, a localized insertion of peptidoglycan precursors in the cylindrical cell wall and a complete new synthesis of the cell poles were observed by De Pedro *et al*. (1997). Because of its dual localization, MurG is probably essential for both the poles and the cylindrical part of peptidoglycan synthesis. A number of MurG 50–20 foci per average cell could be estimated. With approximately 1200 MurG molecules per cell of which about 1000 are membrane bound (see Fig. S3), each focus could contain between 20 and 50 MurG molecules. Therefore, the random disposition of MurG in the cell envelope seems to concur with the idea that the insertion of precursors in the lateral wall occurs at a limited number of discrete but probably dynamic sites.

In the temperature-sensitive strain of MurG (GS58) an even distribution of MurG in the cytosol was visible. The fact that most of these cells underwent lysis after 2 MDs at 42°C emphasizes the involvement of MurG in the synthesis of peptidoglycan.

Prior to division, a mature divisome has to be assembled at the middle of the *E. coli* cell. This assembly is believed to be a multistep process. Previously, Aarsman *et al*. (2005) reported on that the recruitment of the divisome components follows a two-step model: the first step involved the formation of the Z-ring by polymerization of FtsZ at the constriction site and simultaneous localization of ZipA, FtsA and ZapA. During the second step FtsQ, FtsL/B, FtsW, PBP3, FtsN and AmiC are recruited to the constriction site.

The mid-cell localization of MurG was shown to be dependent on the presence of the mature divisome as revealed by the results of the localization studies performed with the FtsZ84(Ts) LMC509, FtsQ(Ts) LMC531, PBP3(Ts) LMC510 strains, and with the wild-type LMC500 cells in the presence of PBP3 inhibitor aztreonam. Mid-cell localization of MurG was not observed in the absence of either the first step or the second step proteins. These findings implicate that MurG might be a component of the divisome. This observation supports a model proposed previously by Nanninga (1998) where MurG was considered to be an essential part of the divisome. In this model the divisome is thought to be composed of subassemblies connected via FtsZ polymers. In each divisome subassembly proteins from the cytoplasm and periplasm are structurally connected (Nanninga, 1998). In light of this, it can be proposed that MurG provides peptidoglycan lipid II precursors specific for division by being part of the divisome subassemblies. Based on the average number of cell division proteins per cell (Nanninga, 2001), an amount of about 50 subassemblies could be present. This could clarify the intense fluorescence signal of MurG observed at mid-cell.

### Interaction of MurG, MreB and MraY proteins

Using DSP as the cross-linker a high-molecular-weight protein complex; comprising two subcomplexes of approximately 250 kDa and 120 kDa; was identified in *E. coli*. This complex contained both MurG and MreB. The protein complex was also detectable, although in a reduced amount, when DSP was not employed. Taken together, the results suggest that MurG and MreB associate with each other. This was further confirmed by the absence of this complex in cells where *mreBCD* was deleted. In addition, the absence of any unspecific (or background) binding in control experiments provided additional evidence for the specificity of this association.

When IP experiments were undertaken with the MraY–β-lactamase–His fusion protein expressing strain, a main high-molecular-weight cross-linked product was seen. This MurG-containing band was also shown to contain MreB and MraY–β-lactamase–His suggesting the existence of an interaction between these three proteins. As MreB localization was shown not to be dependent on MurG, MraY might be the linking factor of MurG and MreB.

The significance of the formation of this multiprotein complex could be implicated in regulation of lateral peptidoglycan synthesis and thereby of cell elongation. Interaction of MraY and MurG may be essential for consecutive fulfilling of their function in the peptidoglycan assembly. Given the fact that the molecular weight of these three proteins together is about 115 kDa (MurG ≈ 38, MreB ≈ 37, MraY ≈ 40 kDa), it is probable that the 250 kDa complex encompasses (in addition to the possibility that some proteins may form oligomers) other proteins like RodA and PBP2 that are believed to be specifically involved in cell elongation (see the model proposed below).

### Inability of MurG to localize peptidoglycan precursors in the *ΔmreBCD* strain might explain its spherical morphology

Association of MurG and MreB raised the question whether they are dependent on each other with respect to their subcellular localization. To exhibit a helical pattern, localization of MreB was not dependent on the presence of MurG. In contrast, MurG was not able to localize in the Δ*mreBCD* strain. As MurG was able to appear at its position when only MreB was absent, it was apparent that its localization might be dependent on the presence of the MreC and MreD. The dependency of localization of MurG on the presence of the MreCD could indicate that these proteins cooperate during the elongation process in particular the lateral cell wall synthesis. In this regard, the loss of rod shape of *mreBCD* deletion strain could be caused by the loss of MurG membrane localization. As a consequence, this could prevent the localized supply of the lipid II precursor to the peptidoglycan synthesizing machinery involved in cell elongation. However, the absence of MreB led also to altered morphology (spherical cells) where the MreB helix is invisible. This can be explained as follows: because the MreB helical structure is affected by the absence of either MreC or MreD (Kruse *et al*., 2005), it can be postulated that MreB might be involved in the organization of these two proteins in the cell membrane and that this MreBCD complex interacts (cooperates) with other proteins (e.g. MraY and MurG) to provide precursors to build the lateral peptidoglycan, and thereby controlling the topography of cell wall morphogenesis. Provided that MreB is essential for rod shape in *E. coli* and in light of the results found by IP, a potential (direct or indirect) association of MurG with MreB through MraY may exist.

### Model involving the role of MurG in the synthesis of peptidoglycan

Recently, a model in which MreB, C and D form an essential membrane-bound complex that directs longitudinal cell wall synthesis has been proposed for *E. coli* (Kruse *et al*., 2005). Using a bacterial two-hybrid system, association of MreC with both MreD and MreB was demonstrated whereas no interaction of MreB with MreD was found (Kruse *et al*., 2005). In this complex a possible interaction of MreC with multiple PBPs (which are known to add precursors to the existing peptidoglycan by a combination of transglycosylation and transpeptidation reactions), including PBP2 was suggested as was previously reported for *Bacillus subtilis* (Daniel and Errington *et al*., 2003; Van den Ent *et al*., 2006) and *Caulobacter crescentus* (Divakaruni *et al*. 2005). Therefore, the authors proposed a model where the MreBCD complex was predicted to interact with PBP2 and RodA (Kruse *et al*., 2005). In the present work, MurG required the presence of MreCD and not of MreB to find its position. Therefore, it can be reasoned that it might interact with MreCD and hence acts in concert with the MreBCD complex in governing the morphogenesis of *E. coli*.

In conclusion, based on the findings described here and on previous reports (Kruse *et al*., 2005), it can be proposed that the involvement of MurG in the peptidoglycan synthesis of *E. coli* concurs with a two-complex model. In the first complex, which is implicated in cell elongation MurG interacts and/or cooperates with MraY, MreBCD, RodA and PBP2 (Fig. 8). In the second complex implicated in division MurG associates with other cell division proteins like FtsA, FtsQ, FtsW and PBP3. The high-molecular-weight class A PBPs, PBP1A and PBP1B, are essential for peptidoglycan synthesis (they possess transglycosylation as well as transpeptidation activity). PBP1A and PBP1B were proposed to play distinct roles in the synthesis of the cell wall where involvement of PBP1B peptidoglycan synthesis specific for division was suggested (Del Portillo and de Pedro, 1990). In addition, a direct interaction between PBP1B and PBP3 at the division site was recently demonstrated (Bertsche *et al*., 2006) and PBP1A and PBP1B do not form a complex (Charpentier *et al*., 2002). Therefore, it can be speculated that PBP1A might belong to the first complex and PBP1B belong to the second complex of the model. However, further studies are needed to investigate how protein interactions (involving MurG) regulate cell elongation and cell division.

## Experimental procedures

### Strains and growth media

*Escherichia coli* K-12 cells were grown to steady state in glucose minimal medium (GB1) containing 6.33 g of K_2_HPO_4_.3H_2_O, 2.95 g of KH_2_PO_4_, 1.05 g of (NH_4_)_2_SO_4_, 0.10 g of MgSO_4_.7H_2_O, 0.28 mg of FeSO_4_.7H_2_O, 7.1 mg of Ca(NO_3_)_2_.4H_2_O, 4 mg of thiamine, 4 g of glucose and 50 mg of required amino acids, per litre pH 7.0 at 28°C. Due to the use of temperature-sensitive strains, 28°C was used as standard growth temperature. LMC500, LMC510, LMC531 required lysine, PA340, and PA340-678 required arginine, asparagine, glutamine, threonine, histidine and leucine, for growth in minimal medium. LMC509 was grown in a low-salt glucose minimal medium (1/2 GB1), containing the same ingredients as GB1 but differing in the amount of salt, which is 1.33 g of K_2_HPO_4_.3H_2_O and 1.48 g of KH_2_PO_4_ per litre, pH 7.0.

To induce filamentation by inhibiting PBP3, cultures were supplemented with 1 mg l^−1^ aztreonam (ICN Biomedicals, Ohio) (freshly resolved in a saturated Na_2_CO_3_ solution) at an optical density at 450 nm (OD_450_) of 0.025. Prior to harvesting, growth was continued for 2 MDs of the cells in the presence of this antibiotic.

All *E. coli* strains were grown at various temperatures (28°C, 37°C or 42°C) in rich medium containing 10 g bactotryptone, 5 g yeast extract, 5 g NaCl, 15 mmol NaOH per litre (TY). When required (see Table 1) 25–50 μg ml^−1^ kanamycin, 100 μg ml^−1^ ampicillin or 50 μg ml^−1^ chloramphenicol was added to the medium. Absorbance was measured at 450 nm (minimal medium) and 600 nm (rich medium) with a 300-T-1 spectrophotometer (Gilford Instruments Laboratories).

**Table 1:** Bacterial strains.

Strain/plasmid	Relevant genotype	Source
LMC500 (MC4100lysA)	F^-^, araD139, Δ(argF-lac)U169, deoC1, flbB5301, ptsF25, rbsR, relA1, rpsL150, lysA1	Taschner *et al*. (1988a)
LMC509	MC4100lysA, *ftsZ*84(Ts)	Taschner *et al*. (1988a)
LMC510	MC4100lysA, *fts*I2158(Ts)	Taschner *et al*. (1988a)
LMC531	MC4100lysA, *ftsQ*1(Ts)	Taschner *et al*. (1988a)
GS58	F^-^, *his, leu, thyA, deo, ara*(Am), *lac*-125(Am), *galU*42 (Am), *galE, tyrT*[*murG*(Am) *supF*-A81(Ts)	Salmond *et al*. (1980)
BW25113-ΔmraY/pMAKmraYec	MraY-β-lactamase-His tag fusion expressing strain	A. Bouhss
PA340	F^-^*argH1*, *thr*^-^, *leuB*6, *gdH*1, *hisG*1, *gltB*3, *thi*-1, *lacY*1, *gal*-6, *Xyl*-7, *ara*-14, *Mtl*-2, *malA*1, *rspL*9, *tonA*2	Wachi *et al*. (1987)
PA340-678	PA340 *gltB*^+^Δ*mre-678*	Wachi *et al*. (1987)
PA340-678 pMEW1	PA340 *glt*B^+^Δ*mre-678*, Km^R^ pMEW1 expresses MreC and MreD constitutively	M. Wachi
BL21(DE3)/pLysS	F^-^, *ompT*, *hsdsB*, (*rB*^-^, *mB*^-^, *gal*, *dcm*(DE3), Cm^R^	Novagen

### Extraction of MurG and MreB from the IMVs

To study the nature of the interaction between MurG, MreB and the inner membrane of *E. coli*, inside-out IMVs were subjected to a variety of chemical reagents. IMVs were prepared as described by Koppelman *et al*. (2004). IMVs were diluted in buffer L (50 mM triethanolamine acetate pH 7.5, 250 mM sucrose, 1 mM DTT) to a final volume of 50 μl and supplemented with 10 mM EDTA, 2 M NaCl, 6 M urea, or 1% Triton X-100. All treatments were carried out on ice. The influence of pH on the interaction between the proteins and the inner membrane was investigated by incubating the IMVs in 0.1 M Na_2_CO_3_ pH 11.5 in distilled water supplemented with 250 mM sucrose, 1 mM DTT and 0.5 mM PMSF for 60 min on ice. After centrifugation, pellets were resuspended in 25 μl sample buffer.

A sample of 40 μg (final protein concentration) IMV diluted in a final volume of 50 μl buffer L was digested by trypsin (10 U ml^−1^). After incubation on ice for 60 min the digestion was stopped by the addition of 4 vols ice-cold acetone, followed by precipitation of the proteins at −70°C for 30 min. The pellets were resuspended in 25 μl sample buffer. Subsequently, the presence of both proteins after all treatments was assessed by SDS-PAGE and immunoblotting.

### Expression and purification of His-MreB MurG-His and production of the antibodies

To construct the His-MreB and His-MurG fusion, the coding sequence was amplified from the *E. coli* K12 chromosome using the following primers: mreB1 (which introduced an NdeI restriction site overlapping with the *mreB* initiation codon 5′-GGAATTC**CATATG**TTGAAAAAATTTC GTGGC-3′) and mreB2 (which contains a BamHI restriction site 5′-CGC**GGATCCA**TTAC TCTTCGCTGAACAGGT-3′), murG1 (primer A) (5′-AA**GAATTC**ATTAAAGAGGAGAGGTTCACGATGAGTGGTCAAGGAA-3′) (containing an EcoRI site in bold) and murG2 (primer B) (5′-AAGGC**AGATCT**ACAATTACGCCCGGGCAACCCGGC-3′) (containing a BglII site in bold) for expression of MurG (Crouvoisier *et al*., 1999). The MreB PCR product was digested with the corresponding restriction enzymes and cloned into the vector pET28a (Novagen) to yield plasmid pRMV1. Construction of MurG-His generating plasmid pDMC3 was described by Crouvoisier *et al*. (1999). *E. coli* strain BL21(DE3)/pLysS was transformed with the plasmid pRMV1. This strain was grown at 37°C in TY medium supplemented with 50 μg ml^−1^ of kanamycin and chloramphenicol to an OD_600_ of 0.4 and expression of the proteins was induced with 1 mM IPTG. The strain JM109 transformed with the plasmid pDMC3 was grown at the same temperature and medium but in the presence of 100 μg ml^−1^ ampicillin and the induction was carried out with 100 μM IPTG. Growth was continued for 4 h. Cells were harvested by centrifugation and resuspended in ice-cold buffer A (20 mM Tris-HCl, 500 mM NaCl, 5 mM imidazole, pH 7.8). The bacteria for production of His-MreB were lysed by passing twice through a French press at the pressure of 8000 psi and subsequently centrifuged for 30 min at 20 000 *g* at 4°C. The JM109 (pDMC3) bacteria were passed three times in a French press at 1100 psi at room temperature. The proteins recovered in the supernatant were purified by Ni-affinity chromatography using His-Bind resin (Novagen) equilibrated with buffer A. The proteins were eluted with a stepwise increasing concentration of imidazole ranging from 20 to 400 mM in a 20 mM Tris-HCl and 500 mM NaCl, pH 7.8, buffer. His-tagged protein was recovered in fractions eluting between 100 and 400 mM imidazole. Imidazole was removed by gel filtration and pure proteins were obtained. Integrity and purity of the protein was checked by SDS-12% PAGE. The protein concentration of the isolated His-tagged proteins was determined with the BCA reagent kit (Pierce, Rockford, IL, USA) according to the manufacturer's protocol. The fractions with the highest purity were used for rabbit immunization and antibody production. The anti-MreB and anti-MurG sera were affinity-purified (see below) using purified His-MreB or MurG-His protein respectively.

### Affinity purification of antibodies

Antisera against MurG were generated by immunizing rabbits by AgriSera (Sweden) with 0.1 mg ml^−1^ montenide-diluted protein solutions. The antisera were then affinity-purified. In brief, pure protein (10 μg) was loaded on SDS-PAGE and subsequently bound to nitrocellulose membrane. After blocking with 5% skim milk for at least 1 h, the membrane was incubated overnight with 100 μl antiserum at 4°C. Next, the membrane was washed three times with PBS (each time for 5 min) and incubated with 60 μl 0.1 M glycine pH 2.5. After 10 min incubation with vigorous agitation the eluted affinity-purified antibodies were supplemented with 8 μl 1 M Tris pH 9.5 to neutralize the pH and 5% BSA.

### Construction of the MraY–β-lactamase–His fusion

The *E. coli mraY* gene in fusion with β-lactamase gene was amplified from plasmid pAYEB16 (Bouhss *et al*., 1999) by PCR with the primers 5′-AGGA**ACATGT**TAGTTT GGCTGGCCG-3′ and 5′-GTC**GGATCC**AAGCTTAATGGTGATGGTGATGGTGCCAATG CTTAATCAGTGAGGC-3′ using the Expand high fidelity polymerase system (Roche Applied Science). The PCR product was cut with BspLU11I and BamHI (in bold) and inserted into the compatible sites NcoI and BamHI of the pET28b vector (Novagen), generating plasmid pETAYEB. In this construct the *mraY* gene was expressed under control of a strong IPTG-inducible promoter, and the encoded MraY-β-lactamase protein carried a C-terminal His_6_ extension. This construct was verified by DNA sequencing and then transformed to a strain deleted from the *mraY* gene to yield the strain BW25113Δ*mraY*/pMAK*mraY*ec (to be published elsewhere by Bouhss *et al*.)

### Immunofluorescence microscopy

#### 

##### Fixation and permeabilization of the cells

The cells were fixed and permeabilized for immunolabelling essentially as described by Den Blaauwen *et al*. (2003). The cells were incubated with the primary antibody at 37°C for 1 h. Incubation with the secondary antibody Cy3-conjugated Donkey Anti-Rabbit IgG (Jackson ImmunoResearch Laboratories, USA) was carried out at 37°C for 30 min followed by visualization by fluorescence microscope.

##### Image analysis

Before analysis, cells were first immobilized on 1% agarose as described by Koppelman *et al*. (2004). Phase contrast and fluorescence images were obtained using an Olympus BX60 fluorescence microscope, equipped with a CoolSnap-*fx* CCD camera and a Cy3 filter (U-MWG filter, 530–550 nm). Cell length was determined in phase contrast images. The localization and intensity of the fluorescence signal were analysed in the fluorescence images of the bacteria using the program Object-Image 2.13 (http://simon.bio.uva.nl/object-image.html). The profiles were normalized to the same length and then averaged.

### Immunoprecipitation and cross-linking

Immunoprecipitation was performed with magnetic Dynabeads Protein A (Invitrogen) as specified by the manufacturer. To this end, 100 μl of membrane fraction was first treated with 1% Triton X-100 for 1 h on ice, then chemically cross-linked to DSP (Pierce, Rockford, IL, USA) (0.25 mg ml^−1^), unless otherwise stated. After incubation at 4°C for at least 2 h, the reaction was stopped by adding 0.2 M ammonium acetate or 50 mM Tris, pH 7.5. In parallel control experiments were performed where chemical cross-linking was omitted from the procedure (as described above) after subjecting the membranes to Triton X-100.

Protein A beads (50 μl) were added to 10 μl of polyclonal anti-MurG or anti-MreB affinity-purified antibodies and incubated at room temperature for 2 h with rotational mixing. After removal of unbound IgG, this protein A beads suspension was added to the membrane sample and then incubated with rotational mixing overnight at 4°C. Antibody–protein complexes bound to the beads were pelleted by centrifugation and washed three to five times with 1 ml PBS.

Fifty microlitres of SDS-PAGE sample buffer [5 mM Tris-HCl pH 6.8, 1% (w/v) glycerol, 0.001% bromophenol blue] without β-mercaptoethanol was added to the beads and the mixture was incubated at 37°C for 5 min. To cleave the cross-linker, 10% β-mercaptoethanol was included in the sample buffer. After centrifugation the supernatant was analysed by SDS-PAGE and immunoblotting.

### Immunoblotting

Samples were separated on SDS-12% polyacrylamide gel and transferred to nitrocellulose membrane. Membranes were incubated with antibodies at a dilution of 1:10 000 in Tris Buffered Saline containing 50 mM Tris, 150 mM NaCl, 0.05% Tween, pH 7.5 (TBST). The secondary antibody was goat anti-rabbit IgG conjugated to horseradish peroxidase (Bio-Rad Laboratories, France) and used at a dilution of 1:10 000 in TBST. Detection was performed with a chemiluminescent plus kit of Amersham Sciences followed by exposure to Hyperfilm (Amersham Biosciences, UK Limited, England).

### Quantification of MurG by immunoblotting

To determine the number of MurG molecules per cell, *E. coli* was grown to steady state in GB1 medium and harvested by centrifugation. Pellets were then resuspended in sample buffer and analysed on SDS-PAGE. In parallel a concentration range of 4–16 ng of purified MurG protein was made. Cell number was determined by viable count. A serial dilutions of bacteria grown in GB1 medium was plated and the number of colonies after 24 h of growth at 28°C was counted. Proteins of the cell extract and the MurG concentration range were separated by 12% polyacrylamide gel electrophoresis and transferred to a nitrocellulose membrane. The membranes were incubated with anti-MurG antibody solution for 1 h at room temperature. After washing and incubation with horseradish peroxidase-conjugated antibodies, a chemiluminescent plus kit (as described above) was used to visualize the bands. The amount of MurG was densitometrically determined in the cell extracts and was compared with the standard curve made with different amounts of pure MurG protein using the program Object-Image 2.13.

## Supplementary material

The following supplementary material is available for this article:

Fig. S1 Extraction of MurG from IMVs of *E. coli* (LMC500). IMVs were incubated in buffer L (*see Experimental procedures*) and supplemented with the indicated chemical reagents for 1 h on ice. After centrifugation, the pellets were resuspended in sample buffer and analysed by SDS-PAGE and immunoblotting with affinity-purified anti-MurG.

Fig. S2 Standard curve used to estimate the number of MurG molecules per *E. coli* cell. A concentration gradient (4, 8 and 16 ng) of purified MurG was made and analysed on immunoblot. The optical density was determined using the program Object-Image 2.13. The x-axis represents the concentration of MurG. On the y-axis the optical density is given. The results are the average of three independent experiments (SD = 33, *R*^2^ = 0.9728).

Fig. S3 Immunoblot analysis of the soluble and membranebound fractions of MurG in wild-type (WT) *E. coli* and in the Δ*mreBCD* deletion mutant strain. Membrane (M) and cytosolic (C) fractions were prepared from wild-type (LMC500) and Δ*mreBCD* mutant PA340-678 cells. The same amount of proteins from these fractions was analysed on SDS-PAGE. The immunoblot was probed with anti-MurG. In WT cells MurG is membrane-bound. A very faint band is also visible in the cytoplasmic fraction. In the Δ*mreBCD* deletion mutant strain, MurG is mostly detected in the membrane fraction but is also present in the cytolsolic fraction. The ratio of the amount of MurG present in membrane and cytosolic fractions was densitometrically estimated to be 6.8 in the WT and 3.4 in the Δ*mreBCD* deletion mutant cells.

Fig. S4 Localization of MurG in the PBP3(Ts) strain LMC510 (A), FtsQ(Ts) strain LMC531 (B), and in the wildtype *E. coli* strain LMC500 in the presence of aztreonam (C). LMC531 and LMC510 cells were grown to steady state at 28°C in GB1 medium and then shifted to 42°C and grown for 2 MDs. LMC500 cells were grown at 28°C in GB1 after which aztreonam was added and growth continued for 2°MDs. Thereafter all cells were fixed, permeabilized and immunolabelled with antibodies directed against MurG. LMC531 and LMC510 filaments showed no mid-cell localization of MurG, whereas in aztreonam-induced filaments a clear mid-cell localization is visible (see arrows). MurG mid-cell localization was also absent in the FtsW(Ts) strain (data not shown). Scale bar equals 1 μm.

Fig. S5 Assessment of background binding of the secondary antibodies used for the detection of the immunoblots depicted in Fig. 4 (A and B). During this procedure the primary antibodies were omitted from the procedure. As depicted here no protein bands are visible.

Fig. S6 Localization of MreB in wild-type PA340 (A) and in the Δ*mreBCD* mutant PA340-678 (B) cells. As described in *Experimental procedures* these cells were grown to midexponential phase at 28°C in TY medium. Thereafter cells were fixed, permeabilized and immunolabelled with antibodies directed against MreB. In the strain PA340, MreB localizes in a typical helical pattern. This pattern is not visible in the deletion mutant. Phase contrast (left) and fluorescence images (right) are shown. Scale bar equals 1 μm.

Fig. S7 MurG localization is independent of the spherical cell morphology. LMC500 cells were grown for 2 MDs in the presence of mecillinam (inhibitor of PBP2), at 28°C in TY. Cells were immunolabelled with anti-MurG. In these spherical cells MurG localized normally as multiple foci in the cell envelope. Phase contrast (left) and fluorescence images (right) are shown. Scale bar equals 1 μm.

This material is available as part of the online article from: http://www.blackwell-synergy.com/doi/abs/10.1111/j.1365-2958.2007.05851.x (This link will take you to the article abstract).
