# Supplementary material for: The essential peptidoglycan glycosyltransferase MurG forms a complex with proteins involved in lateral envelope growth as well as with proteins involved in cell division in Escherichia coli
Source: Mol Microbiol. 2007 Aug;65(4):1106–21. doi: 10.1111/j.1365-2958.2007.05851.x (PMC2170320; doi:10.1111/j.1365-2958.2007.05851.x)
Supplement: Fig. S1 — Extraction of MurG from IMVs of E. coli (LMC500). IMVs were incubated in buffer L (see Experimental procedures) and supplemented with the indicated chemical reagents for 1 h on ice. After centrifugation, the pellets were resuspended in sample buffer and analysed by SDS-PAGE and immunoblotting with affinity-purified anti-MurG. [file mmi0065-1106-SD1.pdf]

SUPPLEMENTARY FIGURES

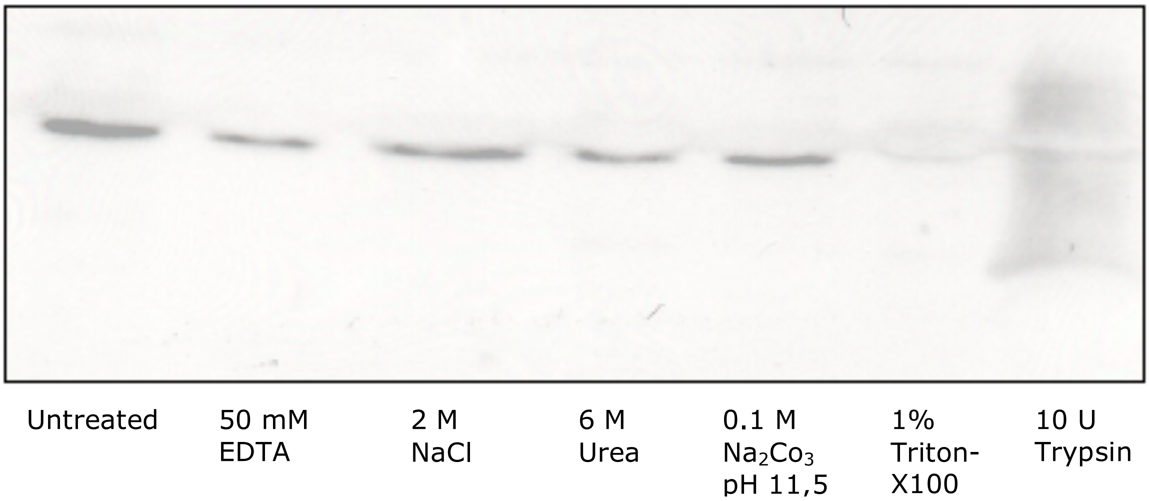

Figure S1: Extraction of MurG from inner membrane vesicles (IMVs) of *E. coli* (LMC500). IMVs were incubated in buffer L (see materials and methods) and supplemented with the indicated chemical reagents for 1 hour on ice. After centrifugation, the pellets were resuspended in sample buffer and analyzed by SDS-PAGE and immunoblotting with affinity purified anti-MurG.

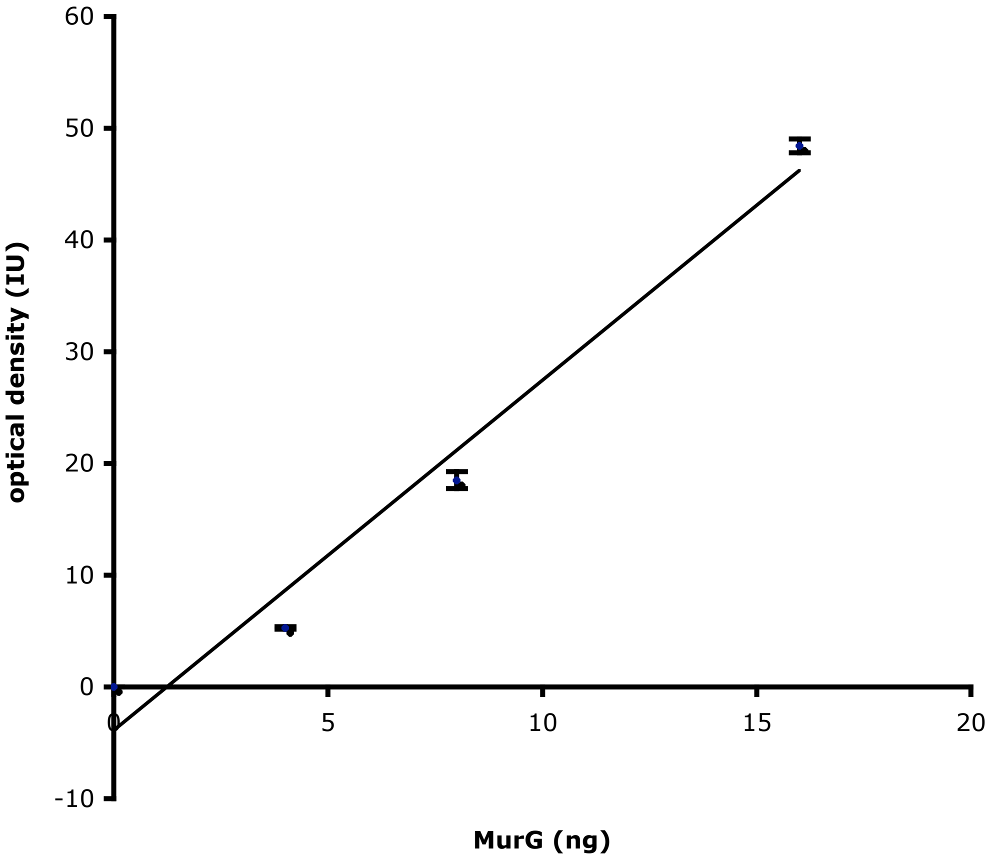

Figure S2: Standard curve used to estimate the number of MurG molecules per *E. coli* cell. A concentration gradient (4, 8 and 16 ng) of purified MurG was made and analyzed on immunoblot. The optical density was determined using the program Object-Image 2.13. The x-axis represents the concentration of MurG. On the y-axis the optical density is given. The results are the average of 3 independent experiments (SD= 33, R^2 = 0.9728).

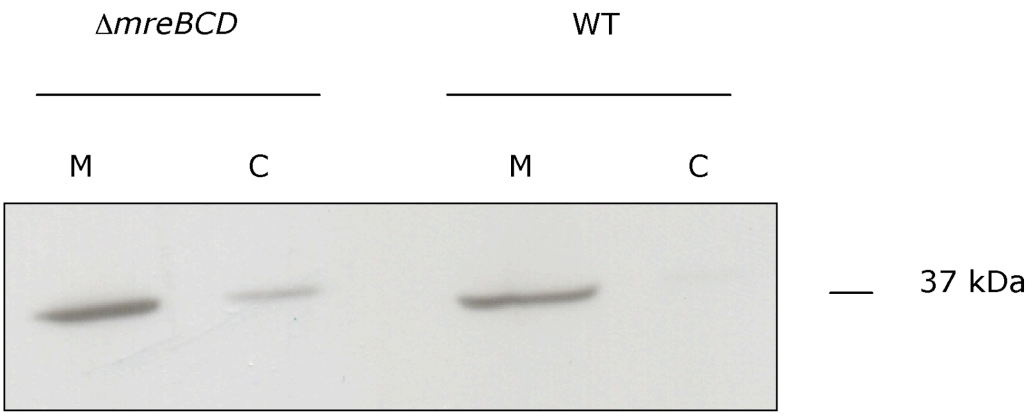

Figure S3. Immunoblot analysis of the soluble and membrane-bound fractions of MurG in wild type (WT) *E. coli* and in the Δ*mreBCD* deletion mutant strain. Membrane (M) and cytosolic (C) fractions were prepared from wild type (LMC500) and Δ*mreBCD* mutant PA340-678 cells. The same amount of proteins from these fractions was analyzed on SDS-PAGE. The immunoblot was probed with anti-MurG. In WT cells MurG is membrane-bound. A very faint band is also visible in the cytoplasmic fraction. In the Δ*mreBCD* deletion mutant strain, MurG is mostly detected in the membrane fraction but is also present in the cytolsolic fraction. The ratio of the amount of MurG present in membrane and cytosolic fractions was densitometrically estimated to be 6.8 in the WT and 3.4 in the Δ*mreBCD* deletion mutant cells.

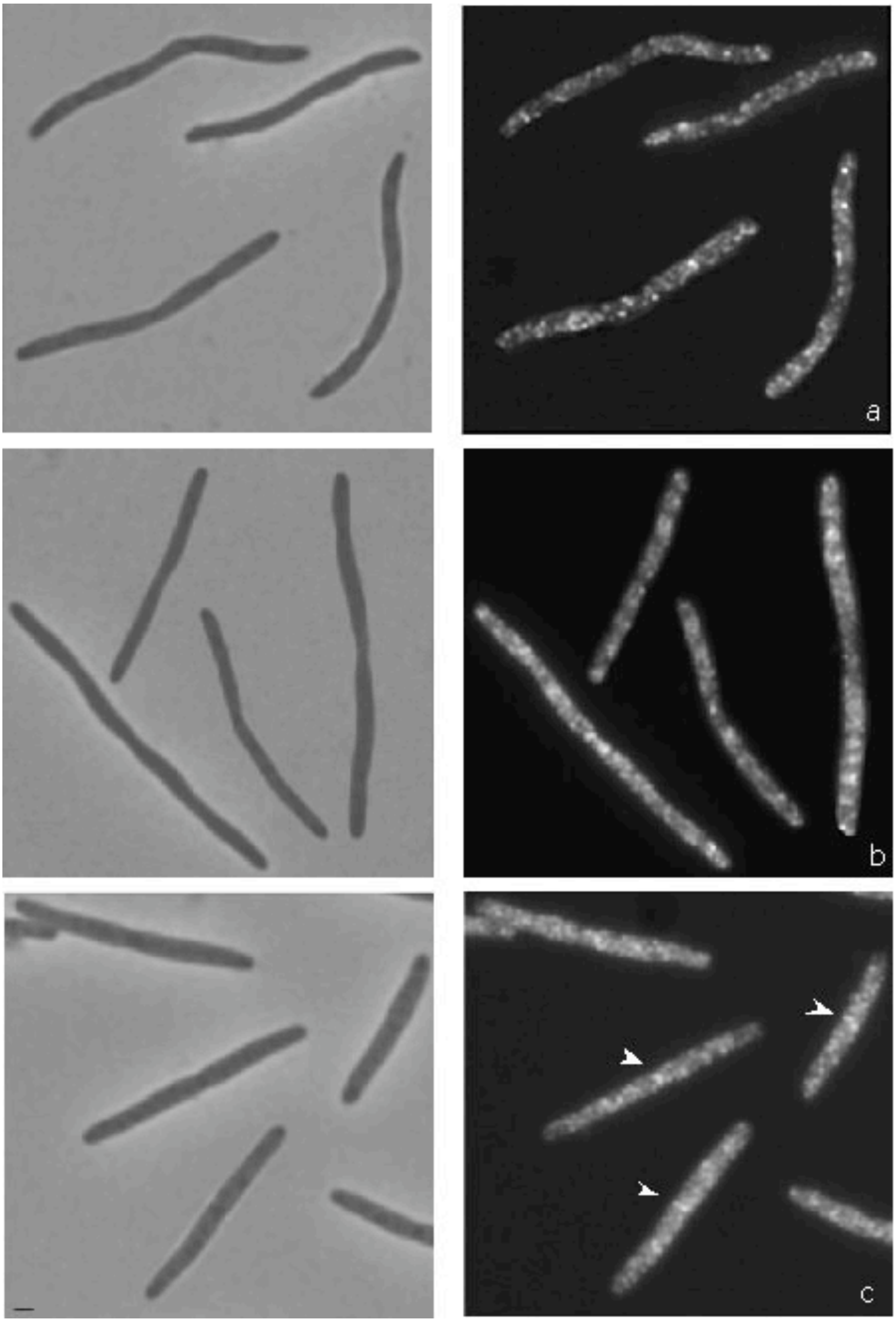

Figure S4. Localization of MurG in the PBP3(Ts) strain LMC510(a), FtsQ(Ts) strain LMC531 (b), and in the wild type *E. coli* strain LMC500 in the presence of aztreonam (c). LMC531 and LMC510 cells were grown to steady state at 28°C in

GB1 medium and then shifted to 42°C and grown for 2 MDs. LMC500 cells were grown at 28°C in GB1 after which aztreonam was added and growth continued for 2 MDs. Thereafter all cells were fixed, permeabilized and immunolabeled with antibodies directed against MurG. LMC531 and LMC510 filaments showed no mid-cell localization of MurG, whereas in aztreonam-induced filaments a clear mid-cell localization is visible (see arrows). MurG mid-cell localization was also absent in the FtsW(Ts) strain (data not shown). Scale bar equals 1 μm.

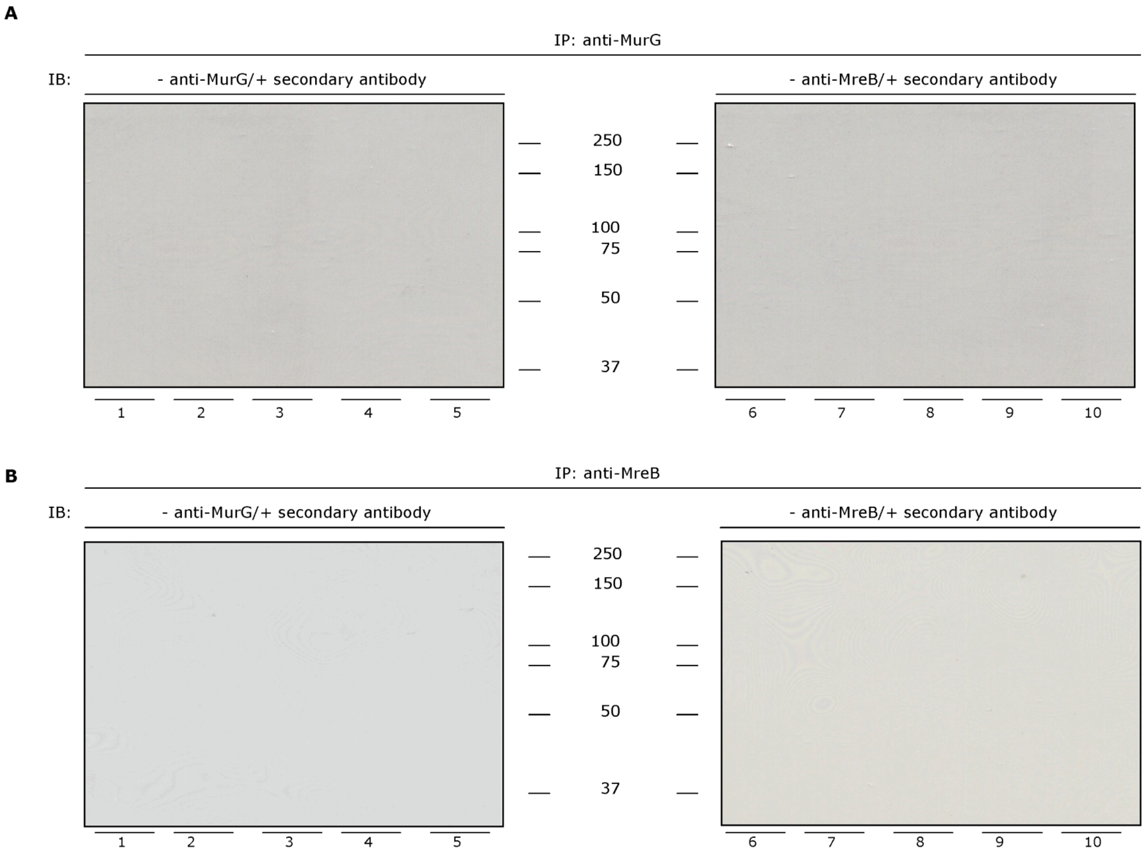

Figure S5. Assessment of background binding of the secondary antibodies used for the detection of the immunoblots depicted in figure 4 (A and B). During this procedure the primary antibodies were omitted from the procedure. As depicted here no protein bands are visible.

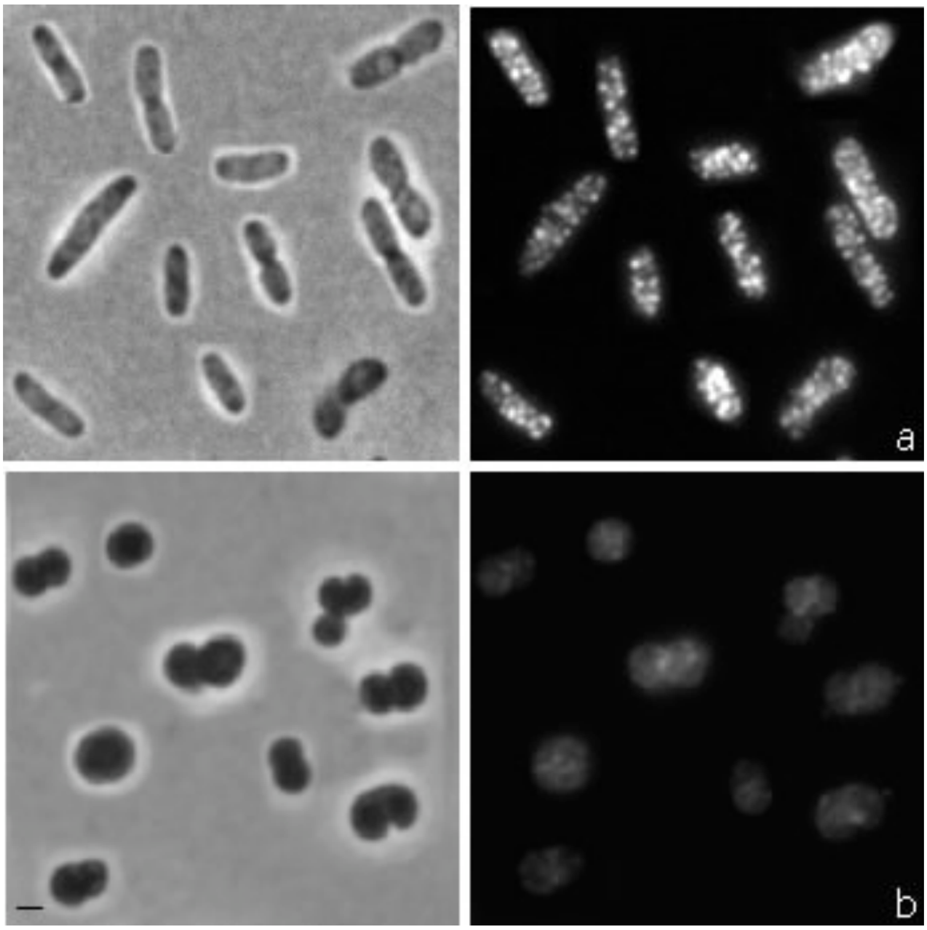

Figure S6. Localization of MreB in wild type PA340 (a) and in the Δ*mreBCD* mutant PA340-678 (b) cells. As described in materials and methods these cells were grown to mid-exponential phase at 28°C in TY medium. Thereafter cells were fixed, permeabilized and immunolabeled with antibodies directed against MreB. In the strain PA340, MreB localizes in a typical helical pattern. This pattern is not visible in the deletion mutant. Phase contrast (left) and fluorescence images (right) are shown. Scale bar equals 1 μm.

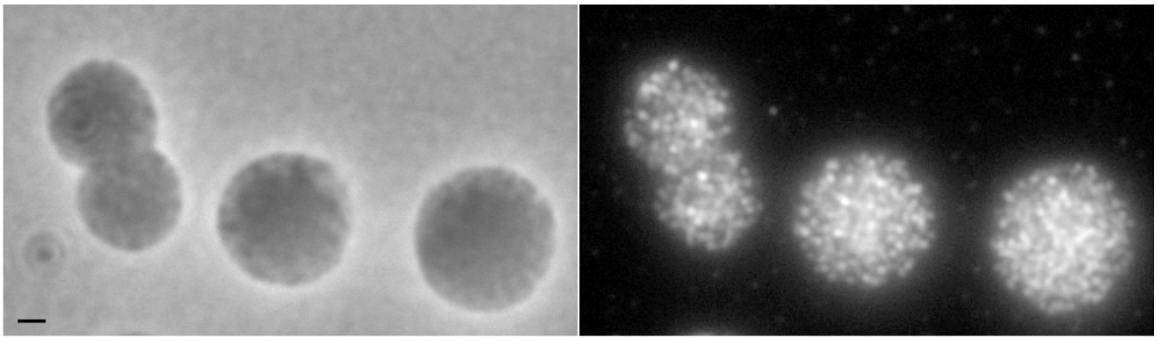

Figure S7. MurG localization is independent of the spherical cell morphology. LMC500 cells were grown for 2MDs in the presence of mecillinam (inhibitor of PBP2), at 28°C in TY. Cells were immunolabeld with anti-MurG. In these spherical cells MurG localized normally as multiple foci in the cell envelope. Phase contrast (left) and fluorescence images (right) are shown. Scale bar equals 1 μm.
